# UHPLC-QTOF-MS/MS based phytochemical characterization and anti-hyperglycemic prospective of hydro-ethanolic leaf extract of *Butea monosperma*

**DOI:** 10.1038/s41598-020-60076-5

**Published:** 2020-02-26

**Authors:** Muhammad Umar Farooq, Muhammad Waseem Mumtaz, Hamid Mukhtar, Umer Rashid, Muhammad Tayyab Akhtar, Syed Ali Raza, Muhammad Nadeem

**Affiliations:** 1 Department of Chemistry, Hafiz Hayat Campus, University of Gujrat, 50700 Gujrat, Pakistan; 2 Institute of Industrial Biotechnology, Government College University Lahore, 54000 Lahore, Pakistan; 3 Department of Chemistry, COMSATS University Islamabad, Abbottabad Campus, Abbottabad, Pakistan; 4 Department of Chemistry, GC University, Lahore, Pakistan

**Keywords:** Metabolomics, Biochemistry

## Abstract

*Butea monosperma* is one of the extensively used plants in traditional system of medicines for many therapeutic purposes. In this study, the antioxidant activity, *α*-glucosidase and *α*-amylase inhibition properties of freeze drying assisted ultrasonicated leaf extracts (hydro-ethanolic) of *B. monosperma* have been investigated. The findings revealed that 60% ethanolic fraction exhibited high phenolic contents, total flavonoid contents, highest antioxidant activity, and promising *α*-glucosidase and *α*-amylase inhibitions. The UHPLC-QTOF-MS/MS analysis indicated the presence of notable metabolites of significant medicinal potential including apigenin, apigenin *C*-hexoside *C*-pentoside, apigenin *C*-hexoside *C*-hexoside, apigenin-6,8-di-*C*-pentoside and genistin etc., in *B. monosperma* leave extract. Docking studies were carried out to determine the possible role of each phytochemical present in leaf extract. Binding affinity data and interaction pattern of all the possible phytochemicals in leaf extract of *B. monosperma* revealed that they can inhibit *α*-amylase and *α*-glucosidase synergistically to prevent hyperglycemia.

## Introduction

*Diabetes mellitus [DM]* is most rapidly growing metabolic disorder in the world. It is primarily characterized by hyperglycemia which is associated with disturbed metabolism of carbohydrates, proteins and fats. Such metabolic dysfunctions at physiological level are known to cause detrimental health disorders which lead towards sickness and eventually death^1^. According to WHO (World Health Organization), it is estimated that this chronic disease has affected nearly 150 million people throughout the world. This number will increase to three hundred million people or more up to 2025^2^. The DM type II (DMT-II) is the most abundant form of diabetes and generally involves the phenomenon of insulin insensitivity or low insulin production. The main reasons behind the spread of this global health problem are mainly modern life style, obesity and consumption of high caloric diet. The growing rate of DM in Asian and African countries is two to three times more than the present rate in other countries^3^. The role of reactive oxygen species (ROS) is very crucial in DMT-II pathogenesis. The ROS are produced because of electron transfer to oxygen from mitochondrial metabolic activity. The ROS are captured by antioxidants to maintain the redox homeostasis. However, over production or long-time exposure to ROS may create imbalance which further leads to state of oxidative stress. The oxidative stress exerts harmful impacts on bio-molecules to create metabolic dysfunction. The ROS under umbrella of oxidative stress disturbs the structure based activity of antioxidant enzymes to reduce the antioxidant potential of body^4^. The ROS are also involved in impaired insulin secretion from pancreas probably due to dysfunction in β-cells^5^. The elevated blood glucose level alters the normal functions of proteins through the process of glycation. The role of glycated end products is obvious in health deterioration and their long term existence may lead to retinal, cardiac, nervous and kidney disorders^6^. Glycated end products also reduce the efficiency of antioxidant enzymes to signify the level of health deterioration^4^. The diabetes initiation or prolongation and its side complication may be controlled or avoided by increasing the antioxidant load. The synthetic antioxidants are available which can be used to eliminate the over production of ROS, cause of oxidative stress. Many effective synthetic drugs are also available to control hyperglycemia. But the toxicity of synthetic antioxidants and harmful impacts of synthetic drugs is a key concern among consumers. The safety issues and toxicity concerns of synthetic compounds are propelling people to consume natural products for disease management. The plant based and herbal medicines are now being consumed by 60% of world’s population^7^. Therapeutic plants can possibly create an enormous assorted variety of anti-oxidative agents. Mechanisms of action, chemical compositions and action sites of these antioxidants are extraordinary different^8^. Antioxidants play a viable inhibitory role in protecting body tissues from damage because of cancer, inflammation and atherosclerosis. They also play an important role so as to avoid unwanted changes in food flavor and nutritional qualities of food^9^. It has already been described in literature that oxidative stress results due to excessive formation of free radicals and due to lack of body’s natural ability to protect itself against these free radicals. This forms the biological basis for many chronic health disorders^10^. Now a days, interest for finding plant based antioxidants for better treatment of chronic ailments is increasing around the globe because of their insignificant or no side effects^11^. Studies concerning the bioactivities of different medicinal plants have gained an imperative position. The metabolite profiling as an essential component of metabolomics is considered as necessary aspect to identify the functional agents responsible for ailment’s cure. Similarly, molecular docking studies also serve as an excellent tool to figure out the binding interactions of plant metabolites with various enzymes to limit their activity. Molecular docking also rationalizes the findings of *in vitro* studies^12^.

*Butea monosperma* (*B. monosperma*) belongs to family *Fabaceae*^13^. This is moderate sized (12 to15 m) tall deciduous tree. Because of its bright red colored papileionaceous flowers, this plant is normally known as flame of forest^14,15^. Its local names are palas, palash, bijasneha, mutthuga, bastard teak, dhak, chichra, khakara and bengalkino. It is common all through India, Burma and Pakistan except in most drastic regions^16,17^. Nearly all plant parts including flower, seed, leaf and bark have curative properties^18–21^.

In traditional system of medicine leaves of *B. monosperma* are used as anti-inflammatory, anti-tumor, diuretic, anti-microbial, anthelmintic, appetite enhancer, carminative, astringent and aphrodisiac. They are also used for the treatment of stomach disorder, sore throat, cough, cold, asymmetrical bleeding during menstruation period and flatulent colic^22^.

Very recent biological studies have confirmed the anti-oxidant as well as antidiabetic potential of some medicinal plants belonging to family Fabaceae, mediated by polyphenols and flavonoid contents^22,23^. Traditionally the leaves of *B. monosperma* in Pakistan are used to treat *DM* but very limited scientific evidence is present in this context. The current work was performed to evaluate the *in-vitro* anti-oxidant and anti-diabetic potential of aqueous, ethanolic and hydroethanolic leaf extracts of *B. monosperma*. The metabolite profiling was performed using ultra high-performance liquid chromatography equipped with quadrupole time of flight and mass spectrometer (UHPLC-QTOF-MS/MS). The binding interactions of identified compounds with carbohydrate hydrolyzing enzymes were also studied by molecular docking.

## Material and Methods

### Extract preparation

Fresh leaves were washed, paper dried, immediately quenched with liquid nitrogen and ground to fine powder. The powder was then lyophilized on Christ laboratory freeze dryer (Germany) at −68 °C under decreased pressure for 48 hours. The crude powder was soaked using ethanol-water solvent systems in different proportions (Pure H_2_O, C_2_H_5_OH 20%-100% with regular interval of 20% in each case) under suitable conditions followed by sonication using 150 Soniprep (UK). All six fractions were then vortexed for about 2 hours at ambient conditions. After filtration excess amount of solvent was evaporated using rotavapor under reduced pressure. Obtained fractions were lyophilized for further analysis at −68 °C for 48 hours. The percent yield of each fraction was calculated and stored at −80 °C for further use.

### Total phenolic contents (TPC)

TPC of understudy extracts were investigated by the Folin-Ciocalteu method^24^. Briefly, 100*µ*L of every sample, after dissolving in CH_3_OH were mixed in 2% Na_2_CO_3_ solution (2 mL). After incubation for 5 min, 100*µ*L Folin Ciocalteu reagent was poured into sample mixture. It was then stayed for 30 min at room temperature (R_T_) for development of color, followed by absorbance measurement at 750 nm through spectrophotometer. Outcomes were articulated as mg of gallic acid equivalent per gram dry extract (mg GAE/g DE)^25^.

### Total flavonoid contents (TFC)

TFC were estimated based upon already reported method^26^. Concisely, 50 mg of each crude sample mixture was soaked in 8mL of aqueous CH_3_OH (80%) followed by filtration using Whatmann no 42-filter paper. After that each sample fraction (300 *µ*L), 30% CH_3_OH (3 mL), 0.5 molar NaNO_2_ solution (125 *µ*L) and 0.3 molar AlCl_3_.6H_2_O solution (125 *µ*L) were mixed. Then further incubated for 5 min and added 1 mL of 1 molar NaOH. Measurement of absorbance was carried out at 510 nm by a spectrophotometer. Standard curve for TPC was drawn using rutin as standard and the results were presented as milligram of rutin equivalent per gram dry extract (mg RE/g DE).

### DPPH radical scavenging assay

Free radical inhibition potentials of the crude extracts were examined via2,2-diphenyl-1-picryl-hydrazil (DPPH) using a previously reported method^27^. Concisely, 1 mL of 0.1 mM DPPH solution in CH_3_OH was added to 3–4 mL of all tested samples. After vigorous stirring the mixtures were kept undisturbed for 30 minutes at R_T_. Then absorbance measurement was done at 517 nm using spectrophotometer (UV-1700, Schimadzu, Japan). The BHA was taken as a standard antioxidant for comparison. The capability to inhibit the DPPH radicals was assessed using the following equation^28^.

$${\rm{DPPH}}\,{\rm{scavenging}}( \% )=\frac{({\rm{Absorbance}}\,{\rm{of}}\,{\rm{control}}-{\rm{Absorbance}}\,{\rm{of}}\,{\rm{sample}})}{{\rm{Absorbance}}\,{\rm{of}}\,{\rm{control}}}\times 100$$

### Total antioxidant power (TAP)

The TAP value is used to assess the total antioxidant capacity of a particular extract or substance. TAP assay was carried out on the basis of reported method with little modification^29^. Briefly, to the 250 µg/mL of each under test extract was added 4 mL of reagent solution (0.6 M H_2_SO_4_ + 4 mM (NH_4_)_2_MoO_4_ + 28 mM Na_3_PO_4_) in plastic vials. The incubation of resulting mixtures and blank was carried out in water bath for 90 minutes at 95 °C followed by subsequent cooling to 25 °C. Absorbance was calculated at 695 nm. Calibration curve was made using ascorbic acid. The anti-oxidant ability was expressed as ascorbic acid equivalent/gram dried extract (ASE/g DE).

### β-carotene bleaching assay (BCB)

Anti-oxidant efficiency of crude plant fractions can also be calculated *in-vitro* by evaluating the bleaching of β-carotene in presence of Linoleic acid. The β-carotene (2 mg) was added in CHCl_3_ (10 mL) along with addition of linoleic acid (0.02 mL) and Tween 40 (0.2 mL)^30^. The 0.2 mL of each crude sample was added in prepared mixture. The positive control (BHA) was also run under same conditions. Then incubation was carried out for 15 min at R_T_ and CHCl_3_ was removed with the help of rotary evaporator at 39 °C followed by addition of 50 mL of H_2_O. The resulting mixtures were vortexed and absorbance was measured before and after incubation for 2 hours at 50 °C.

$${\rm{Antioxidant}}\,{\rm{activity}}( \% )=\frac{1-({\rm{Ao}}-{\rm{At}})}{{\rm{Co}}-{\rm{Ct}}}\times 100$$

where, Ao was absorbance of sample before incubation, Co was the absorbance of control before incubation, At was absorbance of sample after incubation and Ct was absorbance of blank after incubation.

### The *α*-amylase inhibitory activity

The *in-vitro* anti-diabetic potential of each extract was assessed by measuring their inhibition against starch hydrolyzing enzyme, *α*-amylase. For this purpose, about 1% of the sample extract was mixed with potato starch (25 mL) along with continuous stirring. Then the enzyme (100 mg) was added to starch solution, stirred and incubated for 1 hour at 38 °C. After that enzymatic activity was stopped by addition of dinitrosalicylic acid in NaOH (2 mL). The sample mixture was then subjected to centrifugation for a while and glucose contents were calculated in the obtained clear solution. Absorbance was noted at 540 nm spectrophotometrically. A test for positive control (acarbose) was also carried out and percent inhibition was evaluated by formula given below^31^.

$$\alpha -{\rm{amylase}}\,{\rm{inhibition}}\,( \% )=\frac{({\rm{Ab}}-{\rm{As}})}{{\rm{As}}}\times 100$$

where, Ab was absorbance of blank and As was absorbance of Sample.

### The *α*-glucosidase inhibition assay

The *α*-glucosidase inhibitory activity of fractions was performed by following method^32^. The crude hydroethanolic leaf extracts of *B. monosperma* were dissolved in 0.1 molar phosphate buffer (pH = 6.9) containing carbohydrate hydrolyzing enzyme. After incubation at 37 °C for 10 min, reaction was started by adding 10 µL of *p*-nitrophenol-*α*-D-glucopyranoside in buffer. Re-incubation of mixtures was carried out at 25 °C for 5 min and absorbance was noted at 405 nm and compared with acarbose. All the measurements were made in triplicate and percentage inhibition was calculated^33,34^.

$$\alpha -{\rm{glucosidase}}\,{\rm{inhibition}}\,( \% )=\frac{({\rm{Ab}}-{\rm{As}})}{{\rm{As}}}\times 100$$

where, Ab was absorbance of blank and As was absorbance of sample

### UHPLC-QTOF-MS/MS analysis

Metabolites present in 60% hydroethanolic extract of leaves were identified using an advanced analytical technique, UHPLC-Q-TOF-MS/MS. Filtration process was performed by poly-tetraflouroethylene filter having pore size 0.45 µm. The test sample was analyzed by UHPLC quadrupole-TOF-MS/MS (Sciex 5600, provided with Eksigent UHPLC system) and characterized by setting its scanning range from 50–1200 m/z for MS/MS in negative mode of ionization. The Hypersil GOLD UHPLC Column having size 100 mm × 2.1 mm × 3 μm was used. Gradient mobile phase composed of H_2_O and CH_3_CN (Each containing HCOOH (0.1%) & HCOONH_4_ (5 mM)) was used. Gradient elution was carried out from 10% CH_3_CN-90% CH_3_CN with 0.25 mL/min flow rate and injection volume 20 µL. Data interpretation was carried out using Sciex Peak view 2.1 soft-ware, ACD/MS Fragmenter (ACD/Lab) and Chemispider Data-base. Resolved peaks were further identified with the help of reported values from literature.

### Docking studies

Docking studies were carried out by using Molecular Operating Environment (MOE 2016.08). Three-dimensional structure of porcine pancreatic *α*-amylase (PPA) complexed with acarbose was downloaded from Protein Data Bank (PDB code 1OSE). For *α*-glucosidase, docking studies were carried out on homology modelled *α*-glucosidase reported by our research group^35^. Preparation of ligands, downloaded enzymes, 3D protonation, energy minimization and determination of binding site was carried out by our previously reported methods^35,36^. The view of the docking results and analysis of their surface with graphical representations were done using MOE and discovery studio visualizer^37^.

### Statistical analysis

The statistical analysis was performed to evaluate the significance level of difference in means by applying one way Analysis of Variance (ANOVA) through Minitab 17.0 software. The standard deviation (SD±) was also calculated for triplicate values.

## Results and Discussion

### Extract yield

**Figure 1:**
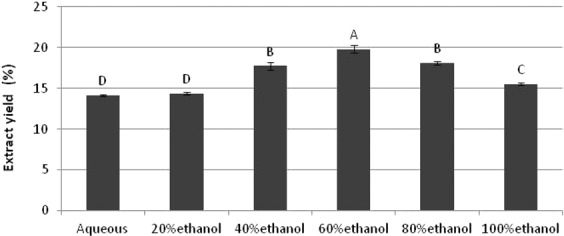
Extraction yield (%) of different hydro-ethanolic extracts.

Yields of aqueous and hydroethanolic extracts of *B. monosperma* are shown in Fig. 1. The different solvent systems selected for extraction influenced the extract yields from leaves. The maximum extract yield (19.79 ± 0.49%) was obtained with 60% ethanol. It was considerably different from extract yields achieved by 100% ethanol (15.53 ± 0.20%) and 20% ethanol (14.37 ± 0.13%). That is why 60% ethanol was considered as a suitable choice for the optimum extract yields from leaves of *B. monosperma*. The statistical analysis revealed that extract yield for 60% ethanol was significantly higher than other fractions (ρ < 0.05).

### TPC and TFC

Plants, both edible and non-edible are rich source of secondary metabolites including phenolic and flavonoids. These metabolites play a vital role in many activities including anti-oxidant activity^38^. Findings regarding TPC and TFC are summarized in Table 1. The results showed that 60% extract exhibited maximum yield of TPC (125.25 ± 1.25 mg GAE/g DE) and TFC (65.15 ± 0.55 mg RE/g DE), respectively. Aqueous extract exhibited lowest yield of TPC (67.85 ± 1.25mg GAE/g DE) and TFC (27.74 ± 0.74 mg RE/g DE), respectively. The TPC and TFC are well known for antioxidant potential to reduce oxidative stress to an acceptable level. The statistical analysis indicated that the TPC and TFC by 60% ethanol for both plants were significantly higher than the other solvent systems used (ρ < 0.05). The solvent polarity played a vital role in enhancing the TPC and TFC yields in respective extracts. The ethanol is comparatively safe and non-destructive solvent for the purpose of extraction. The addition of aqueous phase in ethanol probably the decisive factor to enhance the extraction efficiency which was reflected in form of high phenolic and flavonoid levels.

**Table 1:** TPC and TFC in hydroethanolic leaf extracts.

Sr.#	Plant Extracts	TPC (mg GAE/g DE)	TFC (mg RE/g DE)
1.	Aqueous	67.85 ± 1.25^f^	27.74 ± 0.74^f^
2.	20% Ethanol	78.9 ± 1.33^e^	35.55 ± 0.65^e^
3.	40% Ethanol	95.95 ± 1.05^c^	44.5 ± 0.66^c,d^
5.	60% Ethanol	125.25 ± 1.25^a^	65.15 ± 0.55^a^
6.	80% Ethanol	111.15 ± 1.05^b^	55.5 ± 0.75^b^
7.	100% Ethanol	84.4 ± 0.74^d^	40.14 ± 1.02^d^

### DPPH radical scavenging activity

**Figure 2:**
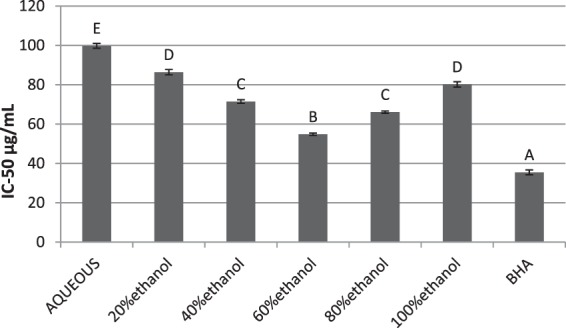
DPPH scavenging potential of extracts of different concentrations.

The potential to scavenge DPPH radical was calculated in terms of IC_50_ value. The IC_50_ value represents the concentration which inhibit a chemical or biochemical process by 50% *in vitro* and is frequently used to express the results of *in vitro* assays^39^. The IC_50_ values for various fractions are presented in Fig. 2. The IC_50_ value of BHA (standard) was computed to be 35.47 ± 1.24 µg/mL. The IC_50_ value of 54.847 ± 0.6 µg/mL was calculated for 60% ethanolic extract followed by the 80% (IC_50_ = 66.08 ± 0.58 µg/mL), 40% (IC_50_ = 71.47 ± 0.92 µg/mL), 100% (IC_50_ = 80.16 ± 1.33 µg/mL) and 20% ethanol extract (IC_50_ = 86.4 ± 1.35 µg/mL). The pure aqueous extract depicted the minimum antioxidant properties as indicated by its IC_50_ value (99.76 ± 1.24 µg/mL). It was concluded that antioxidant potential of all the extracts might be due to high phenolic and flavonoid contents as polyphenols are well recognized natural antioxidants^40,41^. The IC_50_ value of DPPH scavenging by 60% ethanolic extract was significantly higher than the remaining fractions (ρ < 0.05). However, no extract could match the IC_50_ value exhibited by BHA as shown by statistical analysis (ρ < 0.05). The DPPH radical scavenging is widely adopted method to evaluate the antiradical potential of plant extracts. The DPPH scavenging shown by 60% ethanolic extract was comparable to the most recently reported inhibition of aqueous extract of *Strychno spotatorum* (IC_50_ = 50.22 ± 2.21 μg/mL) but less than crude methanol extract of *Adiantum capillus* (IC_50_ = 39.02 μg/mL)^42,43^. The results depicted the 60% ethanolic extract as the most potent antioxidant fraction.

### TAP assay

**Figure 3:**
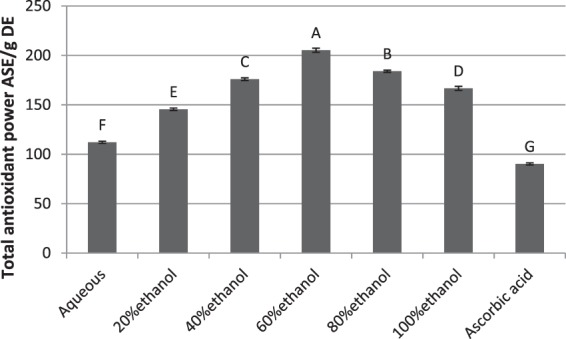
Total antioxidant power assay to assess anti-oxidant activity.

Antioxidant potential of crude leaf extracts was judged by noting the variation in oxidation state of molybdenum (Mo) from +6 to +5 by extracts. This reduction resulted in formation of green color complex which absorbed at 695 nm. The results are presented as Fig. 3. The 60% crude ethanolic fraction showed highest antioxidant capacity having TAP of 205.25 ± 2.05 mg ASE/g DE which was considerably higher than the ascorbic acid (90.2 ± 1.1 mg ASE/g DE). The pure water extract exhibited the lowest TAP value (112.15 ± 1.11 mg ASE/g DE) among all extracts. The TAP value of 60% ethanolic extract was statistically significant when compared with other extracts (ρ < 0.05). The anti-oxidant capacity of 60% ethanolic extract of *B. monosperma* was also significantly higher than formerly reported n-butanol extract of *Anchomanes difformis* (90mg ASE/g DE). These results suggested that 60% ethanolic leaf extract of *B. monosperma* was a rich source of antioxidants^44^.

### Beta carotene linoleic acid assay

**Figure 4:**
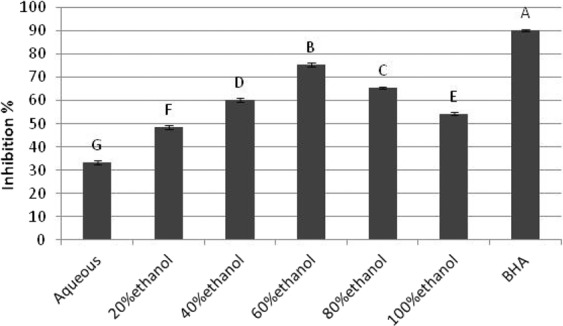
Comparative percentage inhibition for *β-*Carotene bleaching by 60% extracts and BHA.

The peroxide inhibition for bleaching of β-carotene for 60% extract was 75.44 ± 1.05%. Comparative investigation indicated that 60% ethanolic extract exerted the most prominent antioxidant potential among all fractions (Fig. 4). However, no extract could match the inhibition percentage exhibited by BHA (ρ < 0.05). This discriminatory behavior could be because of variable dissemination of bioactives in extracts. The antioxidant capability of 60% ethanolic extract of *B. monosperma* leaves was significantly prominent than recently reported inhibition percentage of *Bromelia laciniosa* ethanolic extract which was 17.88 ± 3.135%^45^. A past report demonstrated that inhibitory potential in bleaching of β-carotene by plant extracts was dose dependent. The high concentrations of extracts might be more effective because of higher contents of bioactive components predominantly phenolic and flavonoids. These were probably responsible for anti-radical and anti-oxidant prospective of plants^46^.

### The inhibition of *α*-amylase and *α*-glucosidase

**Figure 5:**
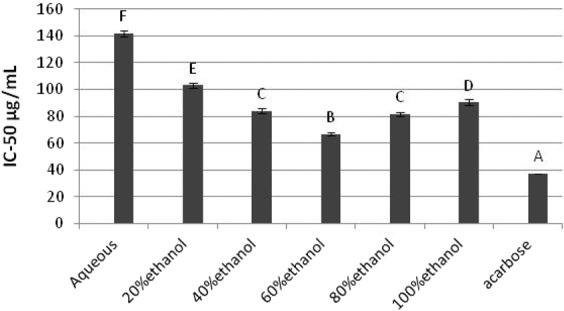
*In-vitro α*-amylase inhibitory potential of *B. monosperma* extracts.

One of the treatment methods to decrease blood glucose level is by inhibiting the carbohydrate hydrolyzing enzymes the, *α*-amylase and *α*-glucosidase^47^. The IC_50_ values for *α*-amylase inhibition are given as Fig. 5. All the extracts exhibited relatively weak inhibitions compared to the acarbose (IC_50_ = 37.16 ± 0.30 µg/mL). The comparative evaluation in terms of statistical analysis determined 60% ethanol as the most effective extract to inhibit *α*-amylase with lowest IC_50_ value of 66.75 ± 1.30 µg/mL (ρ < 0.05). The lowest inhibition of enzyme was shown by aqueous extract as revealed by results (IC_50_ = 141.91 ± 2.175 µg/mL). The *B. monosperma* leaf showed higher inhibition against *α*-amylase than previously reported inhibitory activity of crude ethanolic leaf extract of *Cissus cornifolia* having IC_50_ value of 75.31 ± 9.34 μg/mL^48^.

**Figure 6:**
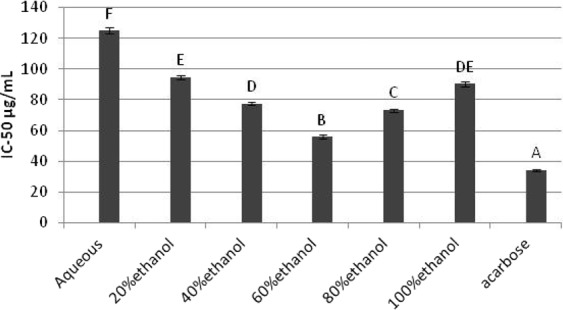
*α*-glucosidase inhibitory potential of *B. monosperma* leaf extracts.

The results of inhibitory effects of *B. monosperma* leaf extracts against *α*- glucosidase are represented as Fig. 6. Enzyme inhibition was influenced by extracts obtained under various solvent compositions designed for extract preparation. The maximum enzyme inhibition was shown by 60% ethanolic extract (IC_50_ = 55.7 ± 1.30 µg/mL) compared to other extracts and found significantly higher than the values exhibited by other extracts (ρ < 0.05). The *α*-glucosidase inhibition potential of 60% ethanolic leaf extract was much higher than previously reported inhibitory activity of ethanolic extract of *Melia azedarach* L. and aqueous extract of *Cissus cornifolia* leaves with IC_50_ values of 3444.11μg/mL and 75.31 ± 9.34 μg/mL respectively^48,49^. The high *α*-amylase and *α*-glucosidase inhibitory properties by hydroethanolic extracts of *B. monosperma* were probably due to presence of some significantly effective phytochemicals. The inhibition of these dietary enzymes by extracts provided an appropriate choice which might be able to low the intestinal glucose absorption leading to decline in postprandial glucose level inside living system^50^.

### UHPLC-Q-TOF-MS/MS analysis

**Figure 7:**
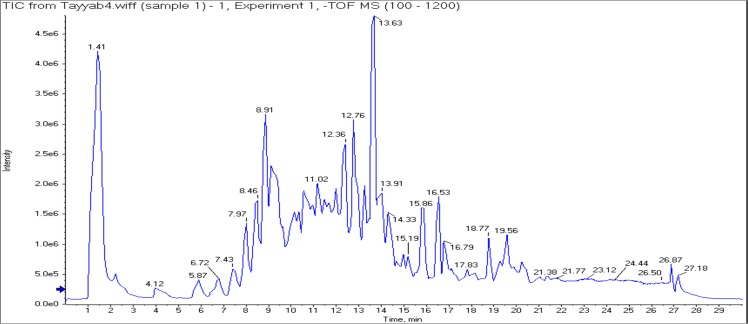
Full chromatogram of 60% extract of *B. monosperma* leaf in negative ion mode.

**Figure 8:**
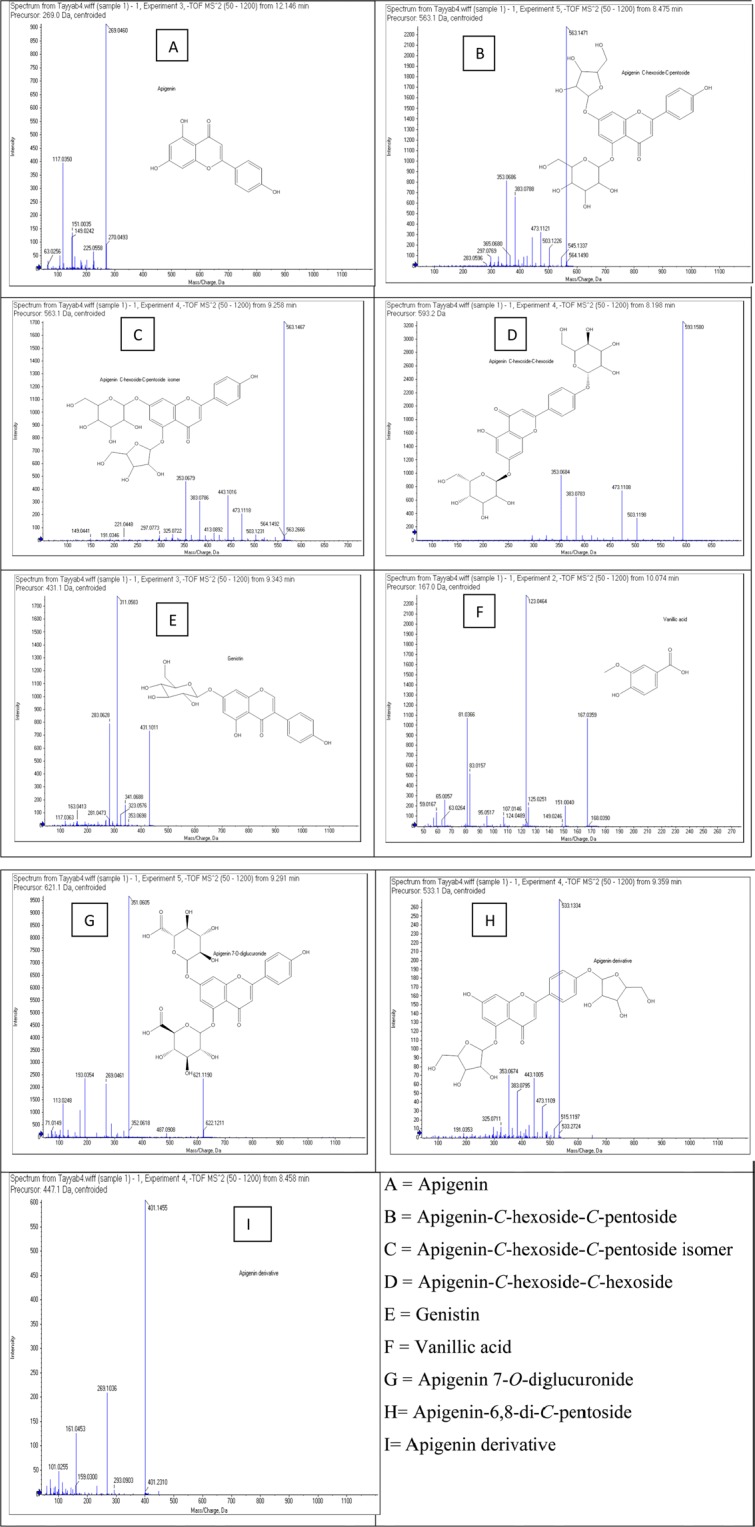
Mass spectra of identified compounds.

UHPLC-QTOF-MS/MS was used for metabolite profiling of 60% ethanolic leaf extract. Full chromatogram of 60% sample is shown as Fig. 7. The mass spectrums along with structures of identified compounds are indicated as Fig. 8. The detail of compounds with their typical fragments (m/z) is given in Table 2.

**Table 2:** Mass spectrometric data of compounds in negative ionization mode.

Sr. #	t_R_ (min)	Molecular Formula	Molecular Weight	[M-H]^−^ (m/z)	Main Fragments(m/z)	Compound
1(a)	12.146	C_15_H_10_O_5_	270.0493	269	225, 151, 117, 63	Apigenin
2(b)	8.475	C_26_H_28_O_14_	564.1490	563	545, 503, 473, 383, 353, 297, 283	Apigenin-*C*-hexoside-*C*-pentoside
3(c)	9.258	C_26_H_28_O_14_	564.1479	563	503, 473, 443, 383, 353	Apigenin-*C*-hexoside-*C*-pentoside isomer
4(d)	8.198	C_27_H_30_O_15_	594.1584	593	503, 473, 383, 353	Apigenin-*C*-hexoside-*C*-hexoside
5(e)	9.343	C_21_H_20_O_10_	432.1056	431	353, 341, 311, 283, 163, 117	Genistein
6(f)	10.074	C_8_H_8_O_4_	168.0390	167	151, 123, 107, 95, 83, 65	Vanillic acid
7(g)	9.291	C_27_H_26_O_17_	622.1211	621	487, 351, 269, 113	Apigenin-7-*O*-diglucuronide
8(h)	9.359	C_25_H_26_O_13_	534.1432	533	515, 473, 443, 383, 353, 325, 191	apigenin-6,8-di-*C*-pentoside
9(i)	8.458	C_19_H_28_O_12_	448.1581	447	401, 293, 269, 161, 101	Apigenin derivative

**Figure 9:**
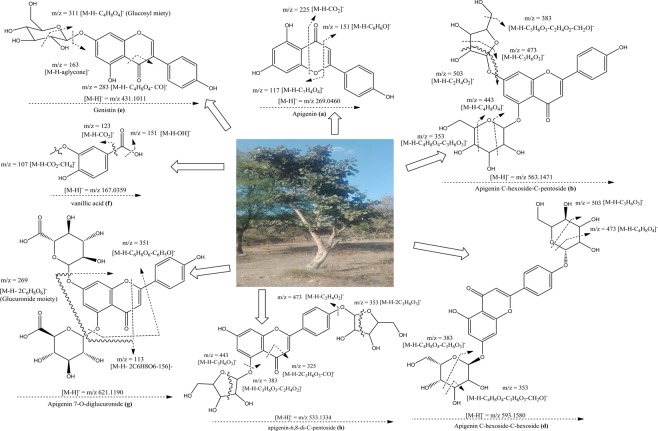
Proposed fragmentation mechanism of bioactives from 60% ethanolic leaf extract of *B. monosperma*.

Proposed fragmentation pattern of the identified compounds are shown in Fig. 9. Compound (1a) was appeared at retention time (t_R_) 12.146 min having molecular ion peak [M-H]^−^ at 269 m/z and its characteristic fragment ion was observed at151 m/z. [M-H-C_8_H_6_O]^−^^51^. Further fragmentation of precursor ion produced daughter ions at 225 m/z and 117 m/z due to neutral loss of CO_2_ and C_7_H_4_O_4_^52,53^ and at m/z 117 in MS spectrum (Fig. 9a). The appearance of these peaks in the chromatogram may be due to cross ring (C-ring)_(C-ring)_ bonds breakage in deprotonated flavonoid molecule^54^ which confirmed compound (1a) as apigenin.

Compounds (2b), (3c) and (4d) were recognized as *C*-glycosylated derivatives . These type of compounds are characterized by the loss of typical fragment loss because of the breakage of sugar pyranos ring, namely −120 amu and −90 amu in case of hexocides^55,56^.

The compounds (2b) and (3c) recorded at the t_R_ 8.475 and t_R_ 9.258 min, respectively contained the common precursor ion [M-H]^−^ at 563 m/z. Further fragmentation generated daughter ion peaks at 473 m/z, [M-H-90]^−^ and at 443 m/z, [M-H-120]^−^ in MS spectrum indicating the existence of cross hexosyl units while the peak at 503 m/z indicated the fragmentation of the pentose sugar. Besides the peaks at 383 m/z and 353 m/z predicted that structures were aglycones of apigenin (Fig. 9b). Consequently, the structures (2b) and (3c) must be apigenin-*C*-hexoside-*C*-pentoside and its isomer^55,57^.

Compound (4d) detected at t_R_ 8.198 min with 593 m/z [M-H]^−^ and a base peak at 353 m/z. Other characteristic peaks were appeared at 503 m/z [M-H-C_3_H_6_O_3_]^−^, 473 m/z [M-H-C_4_H_8_O_4_]^−^and 383 m/z [M-H-C_4_H_8_O_4_-C_3_H_6_O_3_]^−^. The peak at m/z 353 [M-H-2C_4_H_8_O_4_]^−^ was due to apigenin aglycone containing some sugar moieties (270 + 83 amu) linked to it (Fig. 9d)^55,58^. Depending upon the fact that no pertinent ion derived due to complete loss of hexosyl unit (−162 amu) was detected, suggested that sugar was *C-*linked. Thus (4d) was tentatively named as apigenin-*C*-hexoside-*C*-hexoside^56,59^.

Compound (5e) at t_R_ 9.343 min was an *O*-glycosyl flavonoid with molecular ion peak [M-H]^−^ at 431 m/z, producing fragment ion at 311 m/z [M-H-C_4_H_8_O_4_]^−^ due to removal of a glycosyl moiety. It was named as genistein-7-*O*-glucoside (genistin), according to its prior report in *Pterospartum tridentatum*^60,61^. The fragment at 283 m/z was produced due to loss of CO from product ion at m/z 311 [M-H-C_4_H_8_O_4_-CO]^−^^62^. According to our study the peak at 163 m/z might be due to loss of [M-H-268]^−^ (Fig. 9e). This compound was named as genistein-7-*O*-glucoside.

The compound (6f) at t_R_ 10.074 min, having pseudo-molecular ion [M-H]^−^ at 167 was recognized as vanillic acid. On further fragmentation it showed predominant peaks at 151 and 123 owing to successive loss of -OH and CO_2_. The peak [M-H-OH]^−^at 151, m/z [M-H-CO_2_]^−^ at 123 generated same fragment ion at 107 m/z due to neutral loss of CO_2_ and CH_4_ respectively from the precursor ion (Fig. 9f)^63^.

Compound (7g) was detected at t_R_ 9.291 min with precursor ion peak [M-H]^−^ at 621 m/z. Furthermore, two additional peaks were observed in mass spectrum containing one major fragment at 269 m/z and the second minor ion at 351 m/z. The ion at 269 m/z was typical for apigenin aglycone due to neutral loss of the glucuronide moieties [M-H-2C_6_H_8_O_6_]^−^ from the product ion^64^. The other fragment at 351 m/z was observed by loss of the glucuronide moiety and phenyl group [M-H-C_6_H_8_O_6_-C_6_H_5_O]^−^ present in flavone skeleton^65,66^. In view of these perceptions, (7g) was distinguished as apigenin-7-*O*-diglucuronide (Fig. 9g).

Compound (8h) was detected at t_R_ 9.359 min, giving molecular ion [M-H− at 533 m/z as the most intense ion. The base peak at 443 m/z, resulted by neutral loss of 90 amu from precursor ion [M-H-C_2_H_4_O_2_]^−^, which suggested that this compound was C-linked glycoside. Moreover, since mass of deprotonated (8h) was 264 amu more than that of apigenin so it was clearly shown that compound contained two pentose units (132+132amu). Hence (8h) was characterized as apigenin-6,8-di-*C*-pentoside (Fig. 9h)^67,68^.

Apigenin has gained interests since last few decades as a valuable health promoting agent in view of its low inherent toxicity. Apigenin is associated with strong antioxidant and antidiabetic properties. This fact supports the utilization of apigenin rich source in folk medicine for the treatment of DM^69,70^. The methanolic leaf extract of *Achillea sivasica* presented most potent antioxidant properties with IC_50_ 0.22 μg/mL, probably because of the highest phenolic and flavonoid contents including apigenin-*C*-hexoside-*C*-pentoside, apigenin-*C*-hexoside-*C*-hexoside, apigenin-8-*C*-glucoside, coumaric acid hexoside derivative and so forth^71^.

Genistein and two other isoflavone namely daidzein, and glycitin of soybean were previously reported as strong inhibitors of *α*-glucosidase in dose-dependent manner^72^.

The investigation on impact of phenolic acids on glucose uptake was carried out in an insulin resistant cell cultured model. It was reported that vanillic acid improved glucose uptake capacity amongst studied phenolic acids. Moreover, it was reported that a significant decrease occurs in serum insulin level, triglycerides and free fatty acids in rats fed on high fat diet upon consumption of vanillic acid. The study confirmed the protective effect of vanillic acid against hyper-insulinemia, hyperlipidemia and hyperglycemia. These results additionally proposed the capability of vanillic acid in preventing the progress of DM^73^. A recent study reported the antioxidant behavior, *α*-glucosidase and *α*-amylase inhibitory action of *Hyophorbe lagenicaulis* leaf extracts. The phytochemical responsible for the antioxidant and enzyme inhibitory properties in leaf extract of *Hyophorbe lagenicaulis* were identified as kaempferol, rutin, hesperetin 5-O-glucoside, kaempferol-coumaroyl-glucoside, luteolin 3-glucoside, Isorhamnetin-3-O-rutinoside, trimethoxyflavone derivatives and citric acid^74^. Another investigation reported the strong antioxidant and *α*-glucosidase inhibitory potential of apigenin rich leaf extract of *Cycas revoluta*^75^. The findings of current work regarding secondary metabolite identification indicated the high value compounds including apigenin derivatives and vanillic acid, associated with substantial biological attributes.

### Molecular docking studies

**Figure 10:**
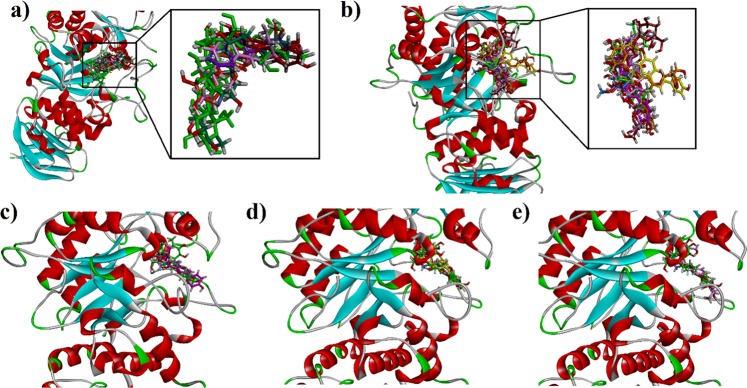
(**a**) The overlaid ribbon diagram of validation set into the binding site of porcine pancreatic *α*-amylase (PDB code 1OSE). (**b**) The overlaid ribbon diagram of isolated phytochemicals into the binding site of porcine pancreatic *α*-amylase; (**c–e**) The overlaid ribbon diagrams of phytochemicals **1**, **3** and **6** and native ligand acarbose in to the binding site of porcine pancreatic *α*-amylase.

To further strengthen our *in vitro* results, we also performed molecular docking studies using Molecular Operating Environment (MOE 2016.08). Before docking studies of phytoconstituents of leaf extract of *B. monosperma*, we performed docking studies on a validation set of the already reported flavones, flavanones and isoflavanone (Table 3). The docking studies on validation set was carried out under the assumption that the predicted binding affinities along with their reported *in vitro* activity for porcine pancreatic *α*-amylase will be predictive of possible role of each phytochemical component in the synergistic effect^76^. Three-dimensional structure of porcine pancreatic *α*-amylase (PPA) complexed with acarbose was downloaded from Protein Data Bank (PDB code 1OSE). For *α*-glucosidase, docking studies were carried out on homology modelled *α*-glucosidase reported by our research group^35^. The binding energy data of the validation set for porcine pancreatic *α*-amylase is given in Table 3. All the compounds are found to show a relationship between binding affinity and IC_50_ value, except for apigenin, which showed weaker binding energy than expected from *in vitro* experiment. The binding cleft of *α*-amylase lies deep near its center and consists of Asp197, Glu233 and Asp300. While, the active site consists of several aromatic residues and side chains. Aromatic residue present are: Trp58, Trp59, Tyr62, His101, Pro163, Ile235, Tyr258, His299, His305 and Ala307. The side chains of Arg61 Asp165, Lys200 and Asp236 are also important. Three-dimensional (3D) binding pose of all superposed compounds of validation set is shown in Fig. 10a. The interaction plot showed that these inhibitors form hydrogen bond interactions with key active site residues as well as residues of the binding cleft. Although, apigenin showed weak binding affinity, it forms hydrogen bonding interactions with Asp197 and Asp300. A hydrophobic π-π stacking interaction was also observed between Trp59 and 4-hydroxyphenyl ring (Fig. 10e). Acarbose (7) with IC_50_ value 5.3 μM and binding affinity of −9.5683 kcal/mol establishes hydrogen bonding interactions with all important residues. Two-dimensional interaction plot of all compounds is shown in Fig. S-1 (Supporting Information).

**Table 3:** Binding affinity data and *in vitro* results of known inhibitors (validation set) of porcine pancreatic *α*-amylase.

Sr. No	Name of compound	Binding affinity	IC_50_ (μM)
**1**	Baicalein	−5.4947	446.4
**2**	Naringenin	−5.4172	450
**3**	Hesperetin	−5.8624	450
**4**	Luteolin	−5.7996	450
**5**	Apigenin	−5.3037	146.8
**6**	Puerarin	−5.2593	394.2
**7**	Acarbose	−9.5683	5.3

**Figure 11:**
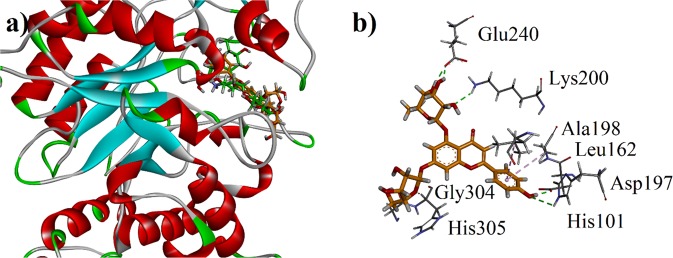
**(a)** The overlaid ribbon diagram of apigenin C-hexoside-C-pentoside (**7)** and acarbose into the binding site of porcine pancreatic *α*-amylase **(b)** Close-up depiction of the lowest-energy three-dimensional (3-D) docking pose of 7.

The bioactive compounds identified through UHPLC-QTOF-MS/MS based phytochemical characterization were subjected to docking simulations to determine their binding affinities. The binding affinity data of the compounds is presented in Table 4. The results showed that binding affinities range from −4.7156 to −9.5683 kcal/mol with porcine pancreatic *α*-amylase. Three-dimensional (3D) binding pose of all the identified bioactive compounds are shown in Fig. 10b. The interactions of the ligands with active site amino acid residues of enzyme are shown in Table 4. Two-dimensional (2D) interaction plot of all compounds is shown in Fig. S-2 (Supporting Information). The Fig. 10c–f showed the binding poses of genistein, apigenin 7-*O*-diglucuronide and apigenin-6,8-di-*C*-pentoside (Compound 1, 3 and 6 in Table 4). The binding-pose of compound 7 (Apigenin-*C*-hexoside-*C*-pentoside isomer, Table 4) superposed on native ligand is shown in Fig. 11a. The 3D binding interaction of establishes hydrogen bond interactions with Asp197, Lys200, Glu240 and Gly304.

**Table 4:** Binding affinity data and ligand interactions shown by possible isolated phytochemicals against porcine pancreatic *α*-amylase.

No.	Compound	Binding Affinity (*α*-Amylase)	Interacting residues of PPA.
1	Genistein	−6.6167	Leu162, Asp197 and Lys200
2	Apigenin	−5.3037	Trp59, Asp197 and Asp300
3	Apigenin-7-*O*-diglucuronide	−8.1976	Glu233, Glu352 and Asp300
4	Vanillic acid	−4.7156	Glu233
5	Apigenin-*C*-hexoside-*C*-hexoside	−8.3671	Gln61, Ile235 and Leu237
6	Aapigenin-6,8-di-*C*-pentoside	−7.6610	Asp197, Lys200, Glu240 and His305
7	Apigenin-*C*-hexoside-*C*-pentoside isomer	−7.3434	Asp197, Lys 200, Glu240, Gly304
8	Acarbose	−9.5683	Trp59, Gln63, Arg195, Asp197, Lys200, His201, Glu233, Glu240, Asp300, Gly306

**Figure 12:**
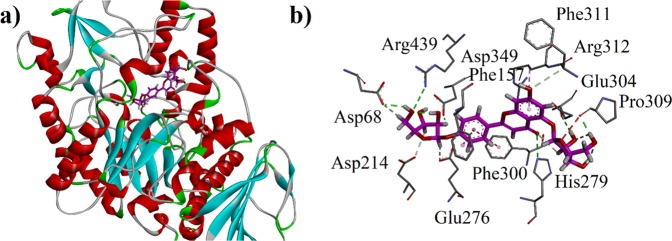
(**a)** Three-dimensional (3-D) docking pose of Aapigenin-6,8-di-C-pentoside (6) into the active site of homology modeled *α*-glucosidase.; (**b**) Close-up depiction of the lowest-energy three-dimensional (3-D) docking pose of **6**.

Docking studies of bioactive compounds against the yeast *α*-glucosidase was carried out on our previously reported homology modelled *α*-glucosidase. Lowest-energy 3D docking pose of Aapigenin-6,8-di-*C*-pentoside (**6)** (Table 4) is shown in Fig. 12a. Compound **6** interacts with Asp68, Phe157, His279, Glu304, Pro309 (Fig. 12b). Two-dimensional (2D) interaction plot of all compounds is shown in Fig. S-3 (Supporting Information). Binding affinity data and interaction pattern of all the possible phytochemicals in leaf extract of *B. monosperma* (Table 5) revealed that they can inhibit *α*-glucosidase synergistically to prevent hyperglycemia.

**Table 5:** Binding affinity data and ligand interactions shown by possible isolated phytochemicals against yeast *α*-glucosidase.

Compound	Binding affinity	Interacting residues
**1**	−7.2759	Asp214, Glu304, Arg312, Arg439
**2**	−5.6631	Asp349, Arg439
**3**	−9.0120	Lys155, Asn241, His279, Arg312, Asn412
**4**	−4.7272	Asp214, Arg439
**5**	−8.2633	Phe157, Asn241, His279, Arg439
**6**	−8.0768	Asp68, His239, His279, Phe300, Pro309, Asp349, Arg439
**7**	−8.1212	Lys155, His279, Arg312, Asp408

The structural interactions of plant based phytochemicals with active sites of α-amylase and α-glucosidase have been reported in some studies. The blockage of active site region of dietary enzymes by secondary metabolites of plants might be a decisive factor behind the enzymatic activity loss. The energy binding calculations regarding activity loss of dietary enzymes reported in previously published literature indicated a close relationship between enzyme inhibition activities of phytochemicals and acarbose^75,77^.

## Conclusions

In current work, antidiabetic and antioxidant potential of hydro-ethanolic leaf extracts of *B. monosperma* were evaluated. The extract yields, TPC and TFC suggested the 60% ethanol as most effective solvent composition for optimum extraction. The 60% ethanolic extract was proved as most efficient fraction with maximum antioxidant and *α*-glucosidase inhibitory potential. The UHPLC-Q-TOF-MS/MS analysis revealed the presence of secondary metabolites of medicinal importance. The findings of molecular docking based on binding affinity data and interaction pattern of phytochemicals in leaf extract of *B. monosperma* revealed that they can inhibit *α*-amylase and *α*-glucosidase synergistically to prevent hyperglycemia.

## Supplementary information

Dataset 1.
