# Supplementary material for: UHPLC-QTOF-MS/MS based phytochemical characterization and anti-hyperglycemic prospective of hydro-ethanolic leaf extract of Butea monosperma
Source: Sci Rep. 2020 Feb 26;10:3530. doi: 10.1038/s41598-020-60076-5 (PMC7044436; doi:10.1038/s41598-020-60076-5)
Supplement: Supplementary file 1 — Dataset 1. [file 41598_2020_60076_MOESM1_ESM.docx]

***Supporting Information***

**UHPLC-QTOF-MS/MS based phytochemical characterization and anti-hyperglycemic prospective of hydro-ethanolic** **leaf extract of *Butea monosperma***

Umer Farooq^1^, Muhammad Waseem Mumtaz^1^*, Hamid Mukhtar ^2^, Umer Rashid^3^, Muhammad Tayyab Akhtar^2^, Syed Ali Raza^4^, Muhammad Nadeem^1^

^1^Department of Chemistry, Hafiz Hayat Campus, University of Gujrat, 50700, Pakistan.

^2^Institute of Industrial Biotechnology, GC University Lahore, Pakistan

^3^Department of Chemistry, COMSATS University Islamabad, Abbottabad Campus

^4^Department of Chemistry, GC University Lahore, Pakistan.

Corresponding Authors Email IDs: [muhammad.waseem@uog.edu.pk](mailto:muhammad.waseem@uog.edu.pk)


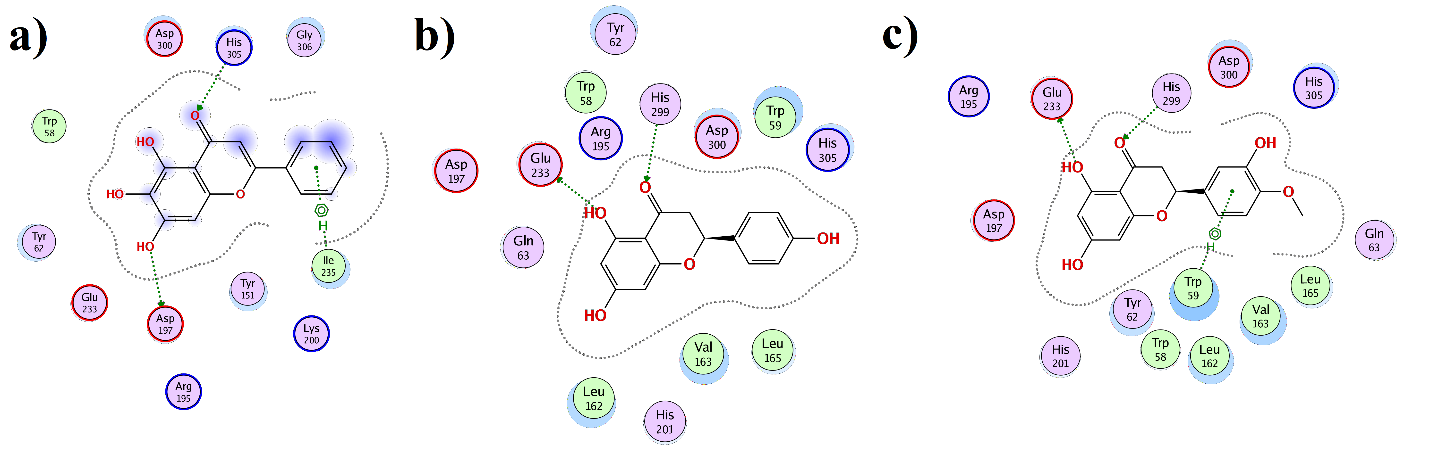


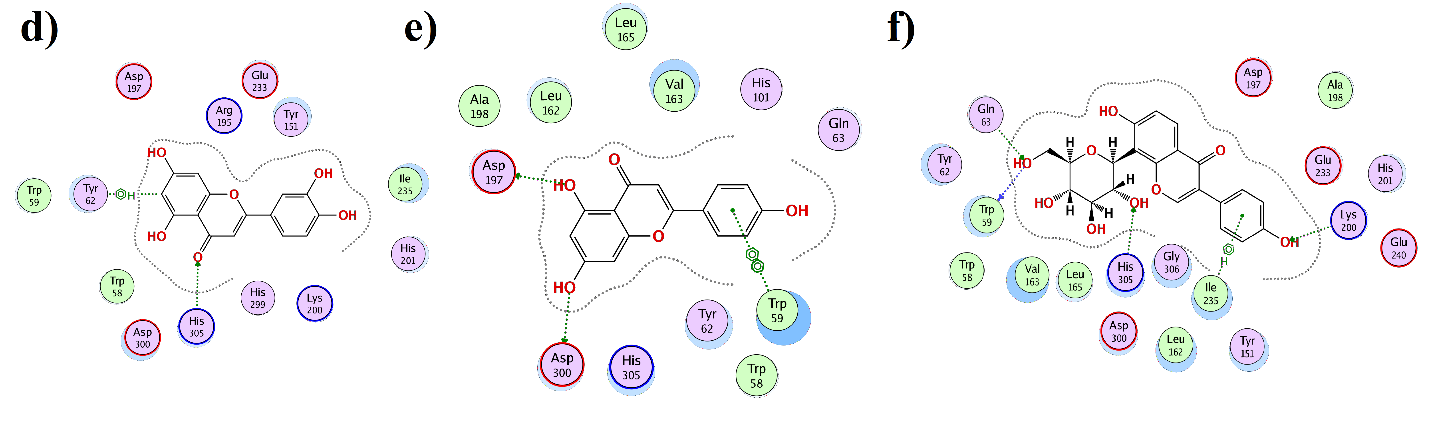


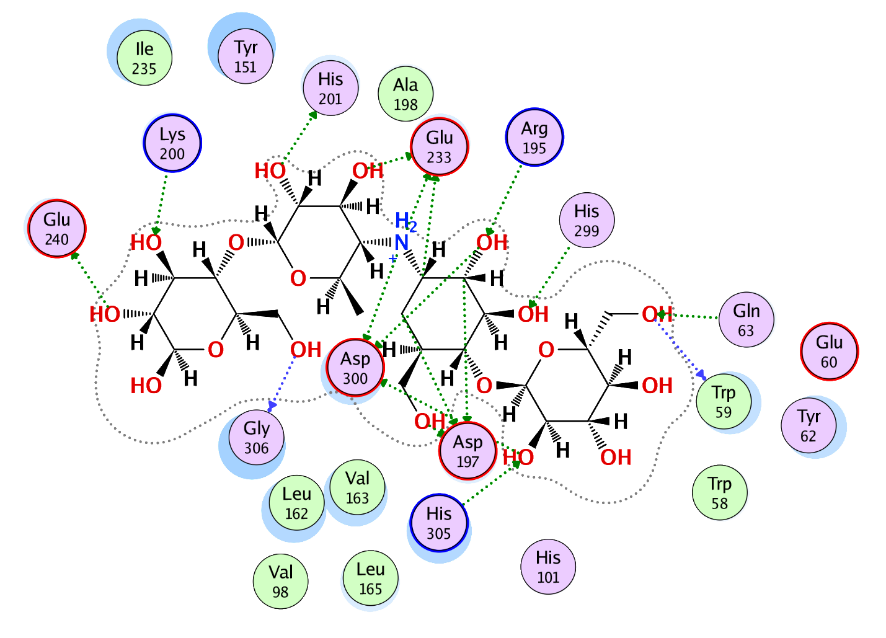


**Figure S-1:** Two-dimensional interaction plot generated by MOE of known inhibitors (validation set) into the binding site of porcine pancreatic α-amylase.


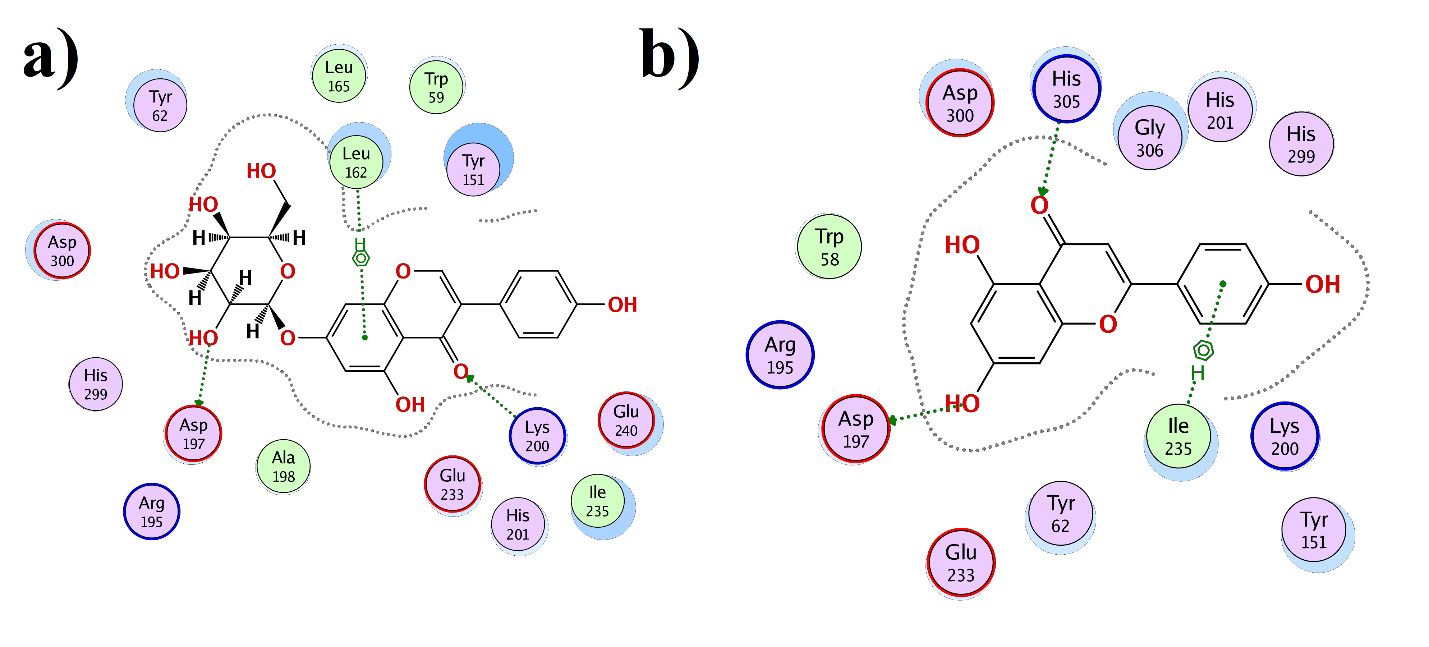


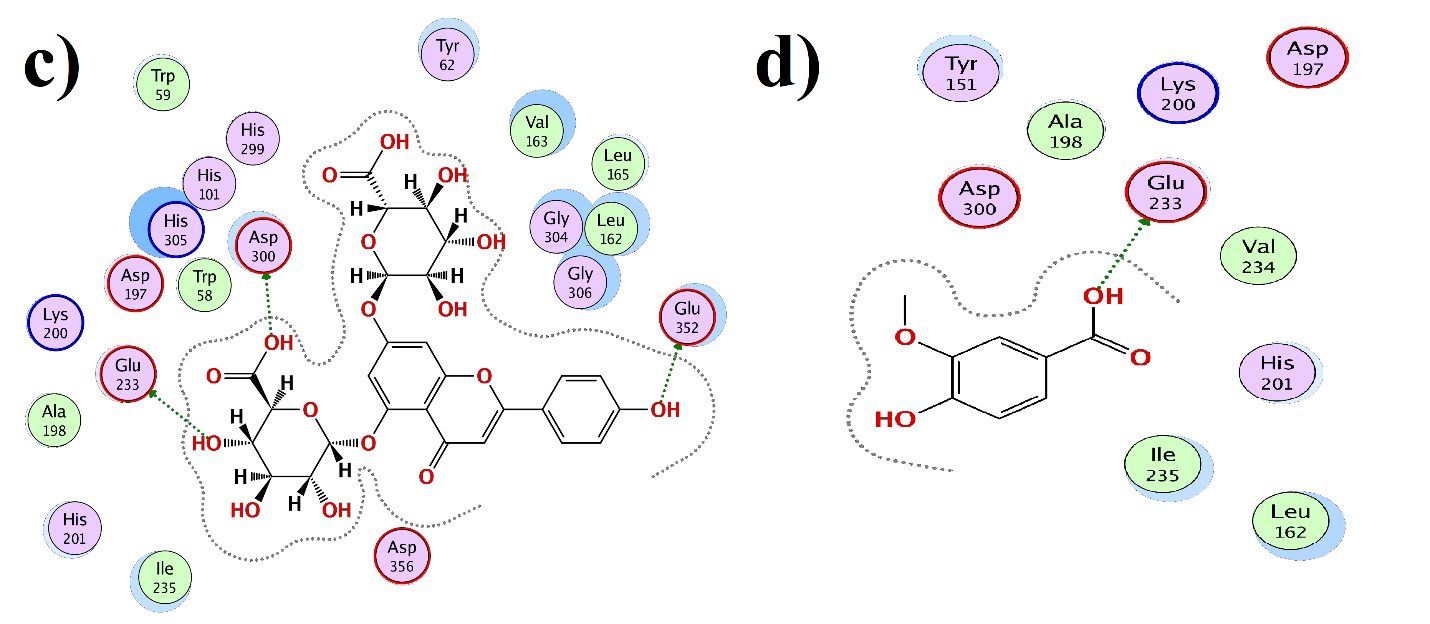


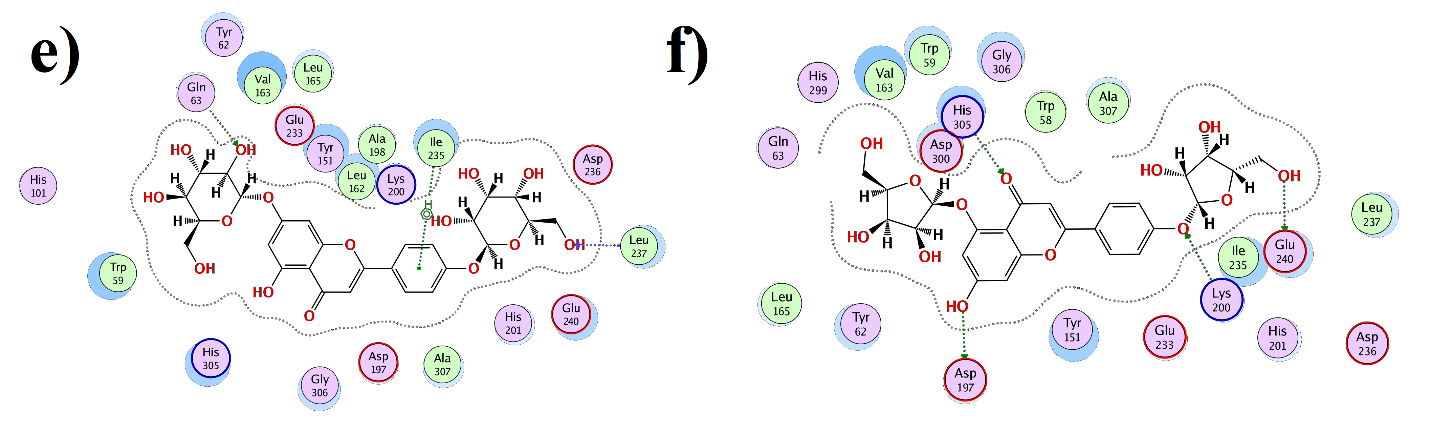


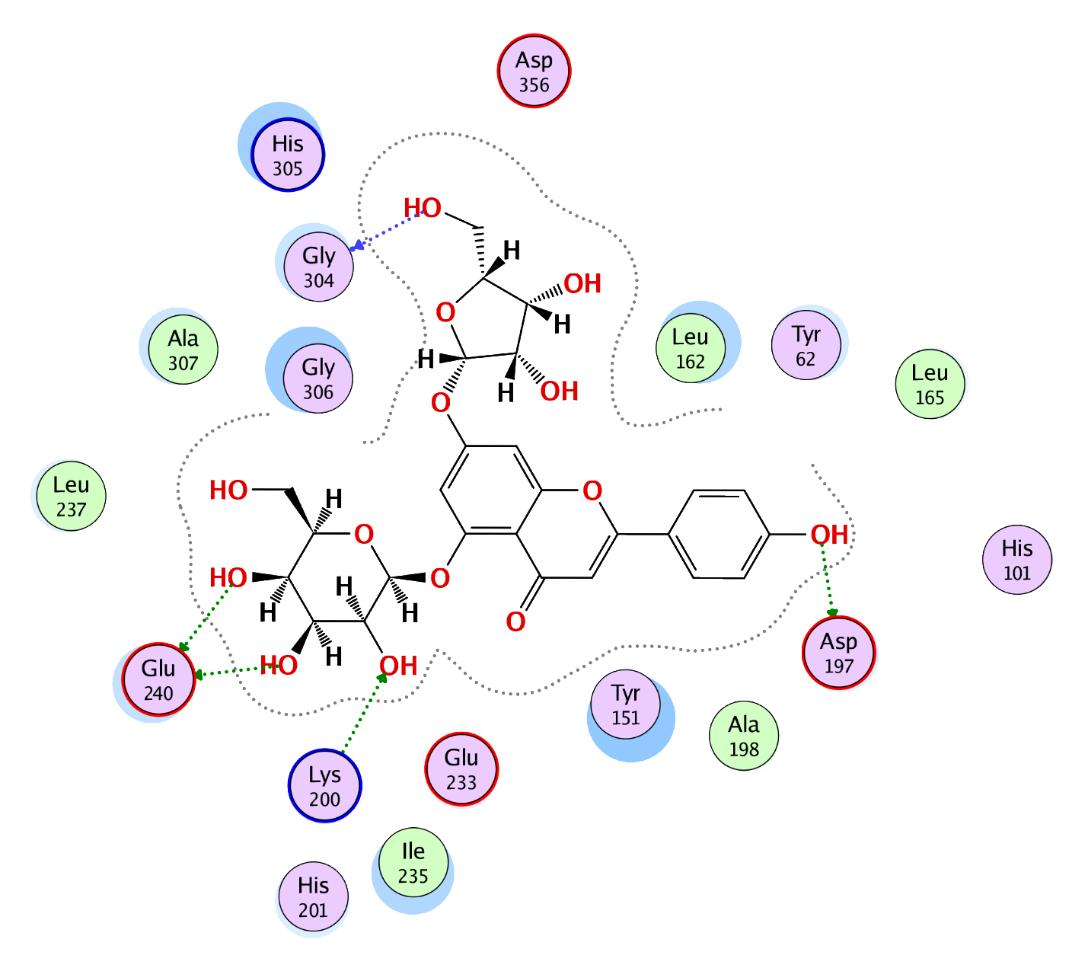


**Figure S-2:** Two-dimensional interaction plot generated by MOE of bioactive phytochemicals from leaf extract of *Butea monosperma* into the binding site of porcine pancreatic α-amylase.


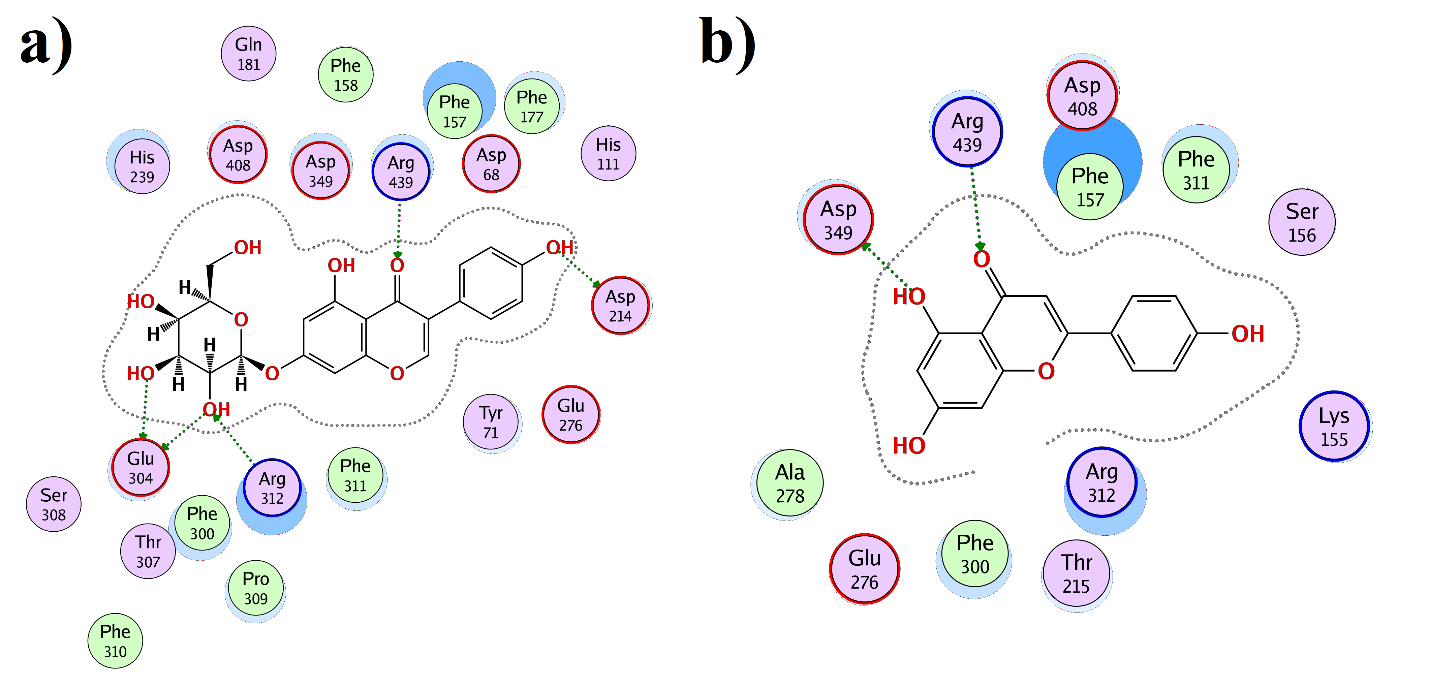


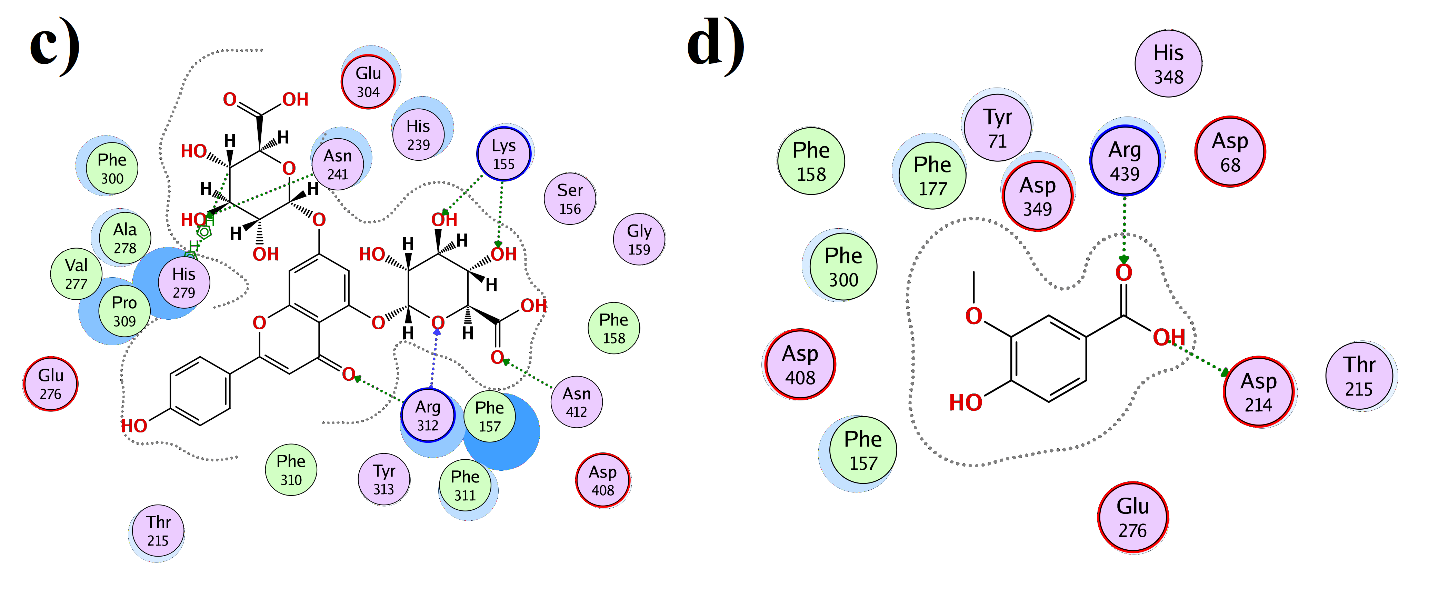


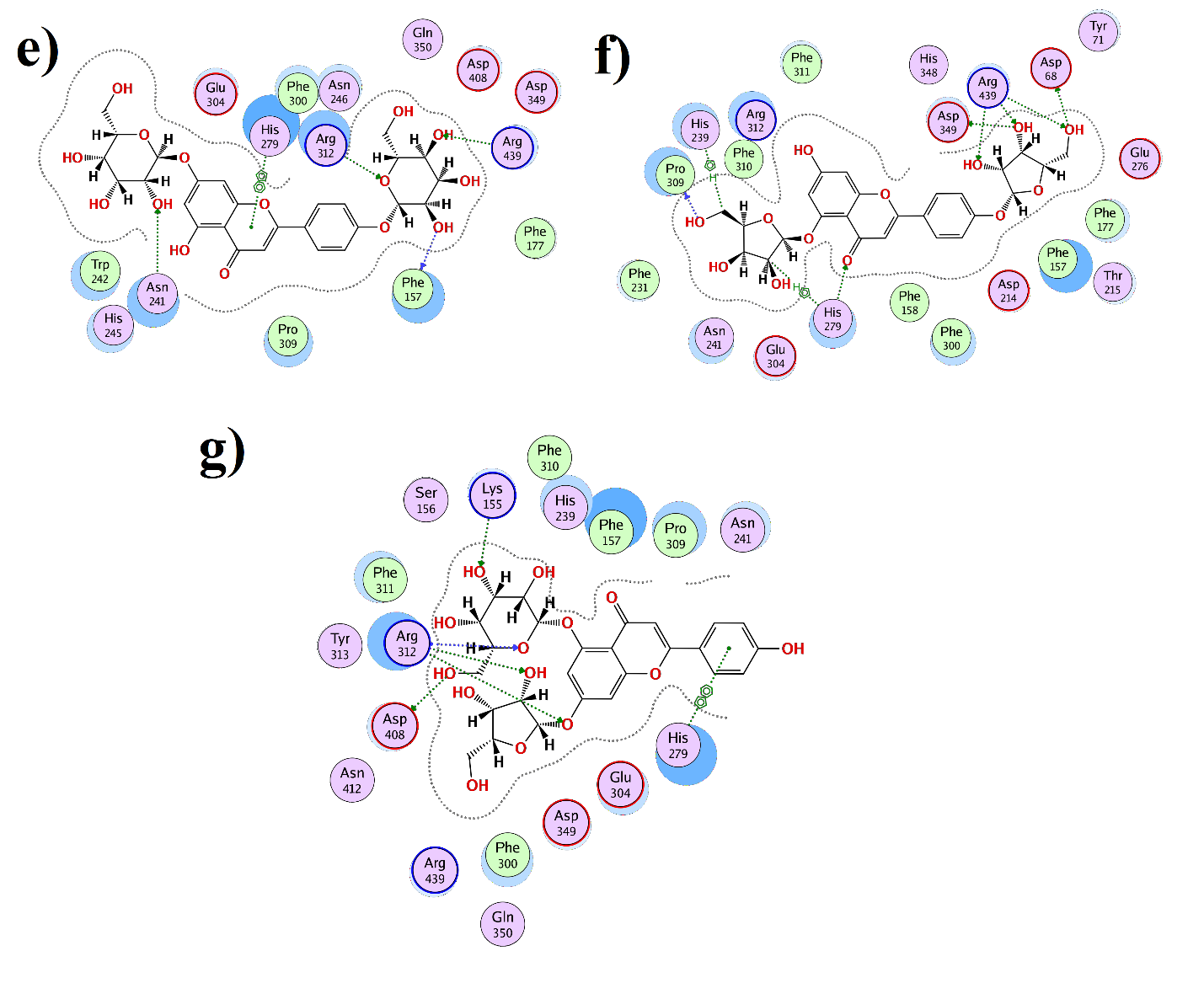


**Figure S-3:** Two-dimensional interaction plot generated by MOE of bioactive phytochemicals from leaf extract of *Butea monosperma* into the binding site of homology modelled α-glucosidase.
